# Rapid and Visually Specific Detection of *Sarcocystis miescheriana* and *Sarcocystis suihominis* Infections in Domestic Pigs (*Sus scrofa domesticus*) Using Loop-Mediated Isothermal Amplification

**DOI:** 10.3390/ani15182763

**Published:** 2025-09-22

**Authors:** Zhun Hu, Tao Qin, Luyao Qian, Lu Xu, Liwu Zhang, Shuangsheng Deng, Jianping Tao, Junjie Hu

**Affiliations:** 1 Yunnan Key Laboratory for Plateau Mountain Ecology and Restoration of Degraded Environments, School of Ecology and Environmental Sciences, Yunnan University, Kunming 650091, China; huzhun@stu.ynu.edu.cn (Z.H.); qintao@stu.ynu.edu.cn (T.Q.); qianluyao@stu.ynu.edu.cn (L.Q.); xulu@stu.ynu.edu.cn (L.X.); 2 Chongqing TopMe Bio-Engineering Co., Ltd., Chongqing 422460, China; zhangliwucq@126.com; 3 Joint Laboratory of Virology & Immunity, School of Biological Sciences, Yunnan University, Kunming 650091, China; ssdeng@ynu.edu.cn; 4 College of Veterinary Medicine, Yangzhou University, Yangzhou 225009, China; yzjptao@126.com

**Keywords:** *Sarcocystis miescheriana*, *Sarcocystis suihominis*, *cox*1, LAMP

## Abstract

The domestic pig (*Sus scrofa domesticus*) serves as an intermediate host for two *Sarcocystis* species: the non-zoonotic *S. miescheriana* and the zoonotic *S. suihominis*, both of which threaten animal and human health and contribute to economic losses in swine production. Existing diagnostic methods, such as microscopy and PCR, suffer from limitations regarding their sensitivity, cost, and field applicability, especially in resource-constrained settings. To address these challenges, we developed a highly specific and ultrasensitive loop-mediated isothermal amplification (LAMP) assay targeting the mitochondrial cytochrome c oxidase subunit I (*cox*1) gene for rapid detection. The optimized protocol (62 °C for 40 min) showed absolute specificity, with no cross-reactivity to related coccidia. The assay exhibited remarkable sensitivity, detecting as little as 6.7 × 10^−6^ ng/μL (*S. miescheriana*) and 5.4 × 10^−7^ ng/μL (*S. suihominis*), representing 10-fold and 10,000-fold improvements over conventional PCR, respectively. With a simple visual readout, the proposed LAMP assay eliminates the need for sophisticated equipment, making it an ideal field-deployable diagnostic tool for basic laboratories and under-resourced regions.

## Simple Summary

Porcine sarcocystosis, a parasitic disease triggered by *Sarcocystis miescheriana* and *Sarcocystis suihominis*, has a widespread global presence among domestic pigs. Nevertheless, it has long been overlooked because of its sub-clinical symptoms in pigs and the lack of obvious macroscopic lesions during routine slaughter inspections. Presently, the diagnosis of sarcocystosis primarily depends on morphological techniques and conventional PCR. Regrettably, both approaches come with significant limitations. Morphological methods are overly reliant on the operator’s expertise and suffer from low sensitivity. On the other hand, the molecular method, i.e., conventional PCR, is a time-consuming process and requires specialized laboratory apparatus. In this study, following the precise identification of the two parasites via morphological and molecular means, we developed a loop-mediated isothermal amplification (LAMP) assay for the rapid detection of sarcocystosis in domestic pigs. LAMP is a well-established isothermal amplification technology, renowned for its simplicity and high-efficiency nature. Additionally, it offers a visually interpretable readout that is easy to understand, rendering it a perfect fit for primary-level laboratories, grassroots disease surveillance initiatives, and regions with scarce resources.

## 1. Introduction

Sarcocystosis is a globally distributed protozoan disease caused by intracellular unicellular organisms of the genus *Sarcocystis*. These parasites exhibit an obligatory two-host life cycle, typically involving herbivores as intermediate hosts and carnivores as definitive hosts. Domestic pigs (*Sus scrofa domesticus*) serve as intermediate hosts for three *Sarcocystis* species: *S. suihominis*, *S. miescheriana*, and *S. porcifelis* [1]. Of these three species, *S. suihominis* and *S. miescheriana* are well-defined morphologically and molecularly, with canids as definitive hosts for *S. miescheriana* and humans/non-human primates for *S. suihominis* [1,2,3]. In contrast, *S. porcifelis*, which has been described only in the former Union of Soviet Socialist Republic (USSR) [4], remains taxonomically controversial due to insufficient morphological and molecular evidence supporting its distinct classification.

Porcine sarcocystosis can cause weight loss, muscle tremors, abortion, and death, depending on the number of sporocysts ingested [5,6]. Human infections with *S. suihominis* exhibit variable clinical manifestations, likely influenced by the number of sarcocysts ingested and host immune responses. For instance, German volunteers who consumed raw pork infected with *S. suihominis* experienced severe symptoms, including bloating, nausea, loss of appetite, stomach ache, vomiting, diarrhea, and tachycardia [7]; in contrast, a Chinese volunteer showed no significant clinical symptoms [8].

Accurate identification of *Sarcocystis* species infecting pigs is critical for controlling its spread and minimizing economic losses. At present, the diagnosis primarily relies on microscopy, histopathological staining of tissue sections, and conventional PCR [1,2]. However, these methods have significant limitations. Microscopy and histopathology are time-consuming, lack sensitivity, and require high levels of technical expertise. Furthermore, although conventional PCR offers better specificity and sensitivity than morphological methods [9], its dependence on sophisticated equipment and trained personnel limits its applicability in resource-limited settings.

Loop-mediated isothermal amplification (LAMP) is a well-established isothermal amplification technique, which employs four to six specific primers targeting distinct regions of the DNA sequence and is known for its high sensitivity and specificity [10]. Due to its simplicity and efficiency, LAMP has been widely adopted for point-of-care testing, genetic diagnostics in resource-limited settings, and rapid screening of food and environmental samples [11,12].

Previous studies have morphologically and molecularly characterized *Sarcocystis* spp. in domestic pigs, identifying the mitochondrial cytochrome c oxidase subunit I (*cox*1) gene as a reliable marker for differentiating *S. suihominis* and *S. miescheriana* [2]. In this study, we developed a LAMP assay targeting the mitochondrial *cox*1 to detect *Sarcocystis* spp. in pigs, providing a rapid and practical alternative for the diagnosis of porcine sarcocystosis.

## 2. Materials and Methods

### 2.1. Sample Collection and Sarcocyst Isolation

Muscle samples (100 g each) were obtained from 57 domestic pigs at a slaughterhouse in Kunming, Yunnan Province, China, between March and June 2024. In the laboratory, ten tissue specimens (approximately 10 × 3 mm each) from each sample were compressed between glass slides and examined under a stereomicroscope (Olympus SZX16, Tokyo, Japan). Sarcocysts were individually isolated from muscle tissues using fine needles for subsequent morphological observation and DNA extraction.

### 2.2. Morphological Characterization and DNA Extraction

Isolated sarcocysts were examined morphologically under a light microscope (Olympus BX53, Tokyo, Japan). For each species, three sarcocysts were individually transferred to sterile 1.5 mL microcentrifuge tubes containing 100 µL of nuclease-free water (ddH_2_O). Genomic DNA was extracted using the TIANamp Genomic DNA Kit (Tiangen Biotech, Beijing, China), according to the manufacturer’s protocol. DNA concentration and purity were assessed using a NanoDrop Lite spectrophotometer (Thermo Fisher Scientific, Waltham, MA, USA), and all DNA extracts were stored at −20 °C for downstream PCR amplification and LAMP analysis.

### 2.3. PCR Amplification

To confirm the morphological identification, the mitochondrial *cox*1 was amplified from six isolates (three each of *S. suihominis* and *S. miescheriana*). PCR amplification was performed in a C1000 Touch Thermal Cycler (Bio-Rad, Hercules, CA, USA) with the universal primers SF1/ SR9 (5′-ATGGCGTACAACAATCATAAAGAA-3′/-5′CCACACCTGTAGTACCDCC-3′) [9,13]. The 25 μL reaction mixture contained 12.5 μL of 2× Taq Plus master mix II, 1 μL of each primer, 9.5 μL of ddH_2_O, and 1 μL of DNA template. The thermal cycling conditions were as follows: initial denaturation at 95 °C for 5 min; followed by 35 cycles of 30 s at 95 °C, 30 s at 55 °C, and 1 min at 72 °C; and a final elongation step at 72 °C for 5 min. The PCR products were then sequenced on an ABI 3730XL automatic DNA sequencer (Applied Biosystems, Foster City, CA, USA).

### 2.4. LAMP Primer Design and Reaction Optimization

#### 2.4.1. Primer Design and Reaction Setup

For the development of a species-specific LAMP assay to distinguish between *S. miescheriana* and *S. suihominis*, the mitochondrial *cox*1 was chosen as the target. The mitochondrial *cox*1 sequences of the two parasites were aligned using BioEdit (Version 7.7.1). Subsequently, primer design was carried out using the NEB LAMP Primer Design Tool Version 1.4.2 (https://lamp.neb.com/). For each species, two sets of primers were generated: outer primers F3/B3, and inner primers FIP/BIP (Table 1). The LAMP reactions were conducted in a 25 μL reaction mixture containing 2.5 μL 10× buffer, 1.5 μL Mg^2+^ (100 mmol/L), 3.5 μL dNTPs (10 mmol/L), 9.5 μL ddH_2_O, 1 μL DNA template, 1 μL Bst DNA 2.0 polymerase (8000 U/L), 2 μL each of the FIP/BIP primers, and 0.5 μL each of the F3/B3 primers. In negative controls, template DNA was replaced with 1 μL ddH_2_O. All reagents were procured from Vazyme Biotech (Nanjing, China). The amplification results were visualized by adding 2.5 μL of 1000× SYBR Green I (Beyotime Biotech, Shanghai, China). Moreover, the reaction solutions were inspected under ultraviolet (UV) light and analyzed via 1.5% agarose gel electrophoresis.

#### 2.4.2. Temperature and Time Optimization

The conditions for the LAMP assay were optimized through two consecutive experiments. In the temperature optimization experiment, the LAMP reactions were incubated at six isothermal conditions (58, 60, 62, 64, 66, and 68 °C) for 60 min. Subsequently, for time optimization, the reactions were carried out for six different durations (20, 30, 40, 50, 60, and 70 min) at 62 °C, which was determined as the optimal temperature in the previous temperature optimization step. All reactions were performed using the standard 25 μL reaction mixture, and ddH_2_O was used as a negative control.

#### 2.4.3. LAMP Specificity and Sensitivity Assay

To assess the specificity of the LAMP assay for the detection of *S. suihominis* and *S. miescheriana*, DNA samples from related coccidia were also analyzed. These included *S. hominis* (of bovine origin), *S. tenella* (of ovine origin), *S. arieticanis* (of ovine origin), *S. medusiformis* (of ovine origin), *Toxoplasma gondii* (of murine origin), and *Eimeria zuernii* (of bovine origin). All samples were tested in triplicate to ensure reproducibility, with ddH_2_O serving as the negative control.

To compare the difference in sensitivity between LAMP and conventional PCR assays, 10-fold serial dilutions (ranging from 1/1 to 1/10^9^) of *S. suihominis* and *S. miescheriana* genomic DNA were prepared. These diluted samples were used as templates for parallel sensitivity testing of the LAMP and PCR assays. The LAMP reactions were conducted using the optimized protocol established through the temperature and time optimization experiments, while the PCR reactions were carried out according to the protocol described in Section 2.3.

## 3. Results

### 3.1. Morphological Observations and Molecular Characterization

**Figure 1:**
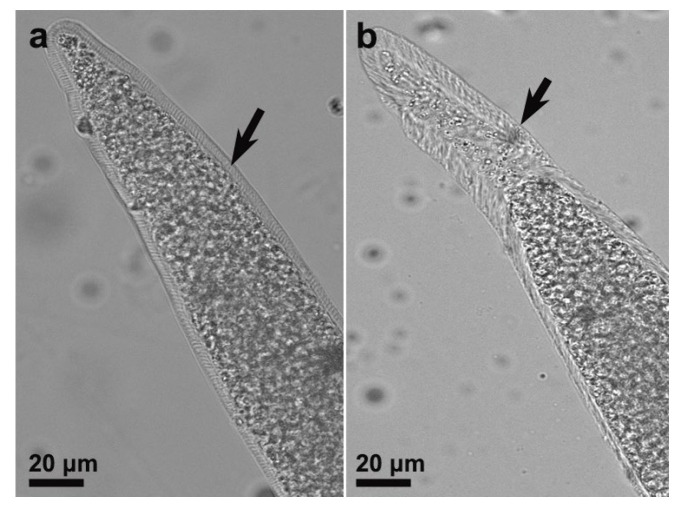
Morphological features of *Sarcocystis* spp. isolated from porcine muscles observed by light microscopy (LM, unstained). (**a**) *Sarcocystis miescheriana* cyst wall showing characteristic short, finger-like protrusions (arrow); (**b**) *Sarcocystis suihominis* cyst wall exhibiting slender, hairlike protrusions (arrow).

Sarcocysts were detected in 31 out of 57 (54.4%) examined domestic pigs. Light microscopy analysis revealed two morphologically distinct *Sarcocystis* species: *Sarcocystis miescheriana* sarcocysts exhibited short, finger-like projections (2.6–5.0 μm in length) oriented nearly perpendicular to the cyst surface (Figure 1a); in contrast, *S. suihominis* sarcocysts displayed slender, hair-like projections that were inclined along the cyst surface (Figure 1b).

Genomic DNA was successfully extracted from individual sarcocysts. The three mitochondrial *cox*1 sequences (1085 bp) obtained for *S. miescheriana* and *S. suihominis* were identical across three isolates for each species. Consequently, a single representative sequence per species was submitted to GenBank (*S. miescheriana*: Accession number PX097380, *S. suihominis*: Accession number PX097381). Comparative analysis revealed 81.6% sequence identity between the two species, confirming their genetic distinction. BLASTn analysis demonstrated that *S. miescheriana* exhibited 98.3–99.6% identity (average 99.2%) with 100% query coverage with respect to reference *S. miescheriana* sequences (GenBank: LC349977–LC349980). Similarly, *S. suihominis* showed 99.2–99.7% identity (average 99.5%) with 65–78% query coverage with respect to published *S. suihominis* sequences (GenBank: OR101956, MK867459–MK867461).

### 3.2. Optimization of LAMP Assay Temperature and Time

**Figure 2:**
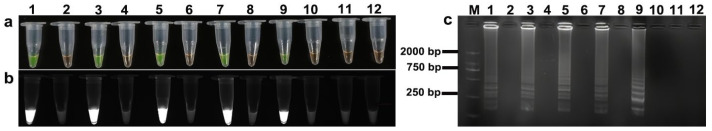
Temperature optimization of *Sarcocystis miescheriana* LAMP assay. (**a**) Visual inspection under natural light; (**b**) fluorescence assessment under UV light; (**c**) agarose gel electrophoresis analysis. Lane designations: M: DL 2000 DNA marker; odd-numbered lanes (1, 3, 5, 7, 9, 11): LAMP reactions conducted at 58, 60, 62, 64, 66, and 68 °C, respectively; even-numbered lanes (2, 4, 6, 8, 10, 12): negative controls (ddH_2_O) at the corresponding temperatures.

**Figure 3:**
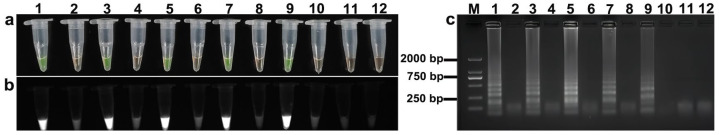
Temperature optimization of *Sarcocystis suihominis* LAMP assay. (**a**) Visual inspection under natural light; (**b**) fluorescence evaluation under UV light; (**c**) agarose gel electrophoresis analysis. Lane designations: M: DL 2000 DNA marker; odd-numbered lanes (1, 3, 5, 7, 9, 11): LAMP reactions conducted at 58, 60, 62, 64, 66, and 68 °C, respectively; even-numbered lanes (2, 4, 6, 8, 10, 12): negative controls (ddH_2_O) at the corresponding temperatures.

The LAMP assay was evaluated at six temperatures (58, 60, 62, 64, 66, and 68 °C) regarding its performance in detecting *S. miescheriana* and *S. suihominis*. Successful amplification was achieved for both species within the temperature range of 58 to 66 °C (Figure 2 and Figure 3). Positive reactions were visualized as follows: yellowish-green fluorescence under natural light (Figure 2a and Figure 3a), fluorescence under UV light (Figure 2b and Figure 3b), and characteristic ladder-like banding patterns on agarose gel electrophoresis (Figure 2c and Figure 3c). No amplification occurred at 68 °C for either species. Negative controls (ddH_2_O) showed no reactivity at all the tested temperatures.

**Figure 4:**
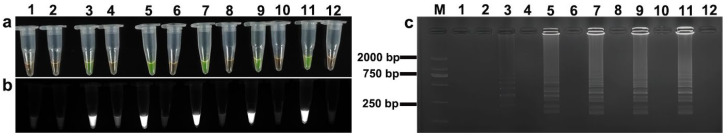
Time optimization of *Sarcocystis miescheriana* LAMP assay incubated at 62 °C. (**a**) Visual assessment under natural light; (**b**) fluorescence evaluation under UV light; (**c**) agarose gel electrophoresis analysis. Lane designations: M: DL 2000 DNA marker; odd-numbered lanes (1, 3, 5, 7, 9, 11): LAMP reactions conducted with 20, 30, 40, 50, 60, and 70 min incubation periods, respectively; even-numbered lanes (2, 4, 6, 8, 10, 12): negative controls (ddH_2_O) at corresponding time points.

**Figure 5:**
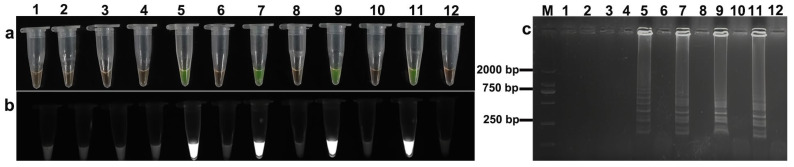
Time optimization of *Sarcocystis suihominis* LAMP assay. (**a**) Visual assessment under natural light; (**b**) fluorescence evaluation under UV light; (**c**) agarose gel electrophoresis analysis. Lane designations: M: DL 2000 DNA Marker; odd-numbered lanes (1, 3, 5, 7, 9, 11): LAMP reactions with 20, 30, 40, 50, 60, and 70 min incubation periods, respectively; even-numbered lanes (2, 4, 6, 8, 10, 12): negative controls (ddH_2_O) at corresponding time points.

To determine the minimal amplification duration, assays were conducted at 62 °C across six time points (20–70 min, 10 min intervals) (Figure 4 and Figure 5). For *S. miescheriana*, positive signals (yellowish-green fluorescence and fluorescence under UV light) were successfully detected from 30 to 70 min (Figure 4a,b). In electrophoresis, faint but detectable bands were observed at 30 min, while the optimal band intensity was achieved after 40 min (Figure 4c). Regarding *S. suihominis*, successful visual and UV detection was achieved from 40 to 70 min (Figure 5a,b), and clear and bright bands were visible when the amplification time was 40 min or longer (Figure 5c). For all the tested time points, no amplification was detected in the no-template control.

### 3.3. Specificity of LAMP Assay

**Figure 6:**
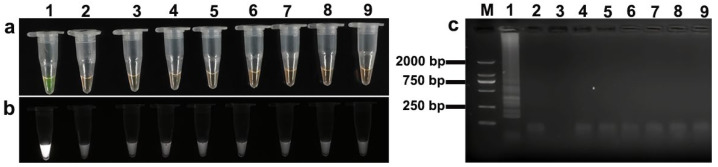
LAMP specificity assay for *Sarcocystis miescheriana*: (**a**) Specificity assessment under natural light; (**b**) specificity assessment under UV light; (**c**) agarose gel electrophoresis specificity assessment. Lane designations: M: DL 2000 DNA Marker; 1: *Sarcocystis miescheriana*; 2: *Sarcocystis suihominis*; 3: *Sarcocystis hominis*; 4: *Sarcocystis tenella*; 5: *Sarcocystis arieticanis*; 6: *Sarcocystis medusiformis*; 7: *Toxoplasma gondii*; 8: *Eimeria zuernii*; 9: negative control (ddH_2_O).

**Figure 7:**
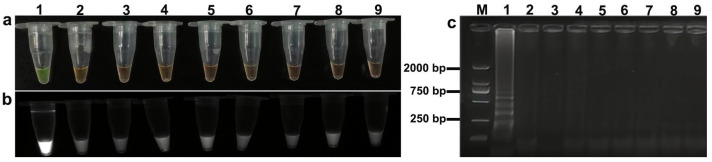
LAMP specificity assay for *Sarcocystis suihominis:* (**a**) Specificity assessment under natural light; (**b**) specificity assessment under UV light; (**c**) agarose gel electrophoresis specificity assessment. Lane designations: M: DL 2000 DNA Marker; 1: *Sarcocystis suihominis*; 2: *Sarcocystis miescheriana*; 3: *Sarcocystis hominis*; 4: *Sarcocystis tenella*; 5: *Sarcocystis arieticanis*; 6: *Sarcocystis medusiformis*; 7: *Toxoplasma gondii*; 8: *Eimeria zuernii*; 9: negative control (ddH_2_O).

The LAMP assay exhibited high specificity towards both target species. In the case of *S. miescheriana* (Figure 6a,b) and *S. suihominis* (Figure 7a,b), positive reactions were characterized by distinct yellowish-green coloration under natural light and clear fluorescence under UV irradiation. Conversely, non-target DNA samples from closely related coccidia showed an orange color under natural light and no fluorescence under UV. These visual cues were further corroborated by the agarose gel electrophoresis results, which revealed characteristic ladder-like banding patterns that were solely present in the reactions containing the DNA of the target species (Figure 6c and Figure 7c).

### 3.4. Sensitivity of LAMP Assay

**Figure 8:**
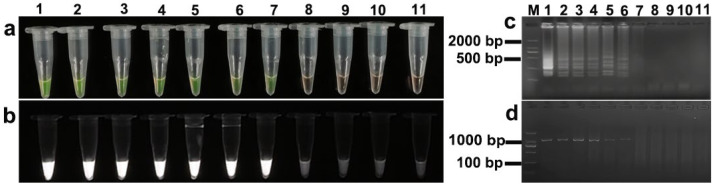
Sensitivity comparison of LAMP and PCR assays for *S. miescheriana* detection: (**a**) LAMP results under natural light; (**b**) LAMP results under UV light; (**c**) LAMP products via agarose electrophoresis; (**d**) PCR products via agarose electrophoresis. Lane designations: M: DL 2000 DNA Marker; lanes 1–10: serial 10-fold dilutions of *S. miescheriana* DNA (from 6.7 ng/μL × 10^0^ to 6.7 ng/μL × 10^−9^); lane 11: negative control (ddH_2_O).

**Figure 9:**
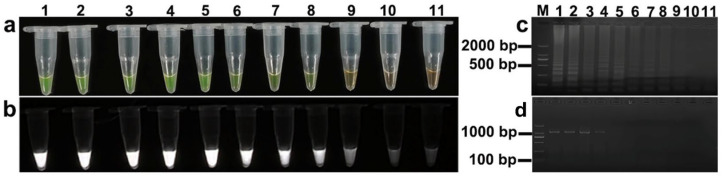
Sensitivity comparison of LAMP and PCR assays for *Sarcocystis suihominis* detection. (**a**) LAMP results under natural light; (**b**) LAMP results under UV light; (**c**) LAMP products via agarose electrophoresis; (**d**) PCR products via agarose electrophoresis. Lane designations: M: DL 2000 DNA Marker; lanes 1–10: serial 10-fold dilutions of *S. suihominis* DNA (from 5.4 ng/μL × 10^0^ to 5.4 ng/μL × 10^−9^); lane 11: negative control (ddH_2_O).

To assess the sensitivity of the LAMP assay relative to conventional PCR, we employed initial DNA concentrations of 6.7 ng/μL for *S. miescheriana* and 5.4 ng/μL for *S. suihominis* as templates. The results clearly indicated substantial disparities in the limits of detection between the two methods. In the case of *S. miescheriana*, through a combination of visualization, fluorescence analysis, and agarose gel electrophoresis, we determined that the LAMP assay could detect DNA at a minimum concentration as low as 6.7 × 10^−6^ ng/μL; conversely, conventional PCR was only able to detect DNA at a minimum concentration of 6.7 × 10^−5^ ng/μL (Figure 8). Likewise, for S. *suihominis*, the LAMP assay demonstrated remarkable sensitivity, with the ability to detect DNA at a minimum concentration of 5.4 × 10^−7^ ng/μL; in contrast, PCR could only achieve detection when the concentration reached 5.4 × 10^−3^ ng/μL (Figure 9).

## 4. Discussion

Sarcocystosis, a parasitic infection caused by *S. miescheriana* and *S. suihominis*, is globally distributed in domestic pigs but remains significantly under-reported. In the field of veterinary medicine, infections with *Sarcocystis* spp. have long been neglected due to their subclinical symptomatic nature in pigs and the absence of obvious macroscopic lesions during routine slaughter inspections [1]. However, emerging evidence reveals alarmingly high infection rates worldwide. Recent studies have reported prevalences of 24.8% (139 out of 561 pigs) in Argentina [3], 36.8% (28 out of 76) in China [2], 31.9% (67 out of 210) in Brazil [14], 23.4% (15 out of 64) in Romania [15], and 58% (29 out of 50) in Malaysia [16]. The present study aligns with this trend, revealing a striking prevalence of 54.4%. In Yunnan Province, China, the practice of free-ranging pig farming and the traditional consumption of raw pork, such as the local delicacy ‘Shengpi’, facilitate the transmission of *S. miescheriana* and *S. suihominis* between domestic pigs (serving as intermediate hosts) and humans or carnivores (acting as definitive hosts). This significantly elevates the risk of zoonotic sarcocystosis, potentially accounting for the high prevalence of the disease in the region.

Most previous studies on detection methods for *Sarcocystis* spp. in pigs have relied on morphological techniques, such as microscopic examination and histopathological staining, and molecular assays, including conventional PCR, real-time quantitative PCR (qPCR), multiplex PCR, and PCR-RFLP [2,3,15,16,17]. However, morphological methods are operator-dependent and suffer from low sensitivity, while molecular techniques are time-consuming and require specialized laboratory equipment. These limitations make it challenging to detect such infections in the field or at the grassroots level in a timely manner. Given these circumstances, there is a clear need for the ongoing development of practical, rapid, and accurate diagnostic tools to enhance the timely detection of sarcocystosis in pigs.

The mitochondrial *cox*1 is highly effective for distinguishing *Sarcocystis* spp. in pigs due to its greater sequence divergence when compared with nuclear rRNA [2]. In this study, combining precise morphological and molecular identification, we developed the first LAMP assay targeting the mitochondrial *cox*1 for detection of *S. miescheriana* and *S. suihominis* in domestic pigs. Since its introduction in 2000, the LAMP assay has emerged as a powerful molecular tool for the diagnosis of microbial and parasitic infections due to its exceptional sensitivity, specificity, and rapidity [10,11,12,18]. Our results confirmed its superior performance, revealing a 10,000-fold increase in sensitivity for *S. suihominis* and 10-fold increase for *S. miescheriana*, compared with conventional PCR, when using the same mitochondrial *cox*1 target. To date, only two studies have applied LAMP assays for the detection of animal sarcocystosis, with differing outcomes regarding sensitivity: Liu et al. (2022) found that LAMP was 1000 times more sensitive than PCR when targeting *S. suihominis* via 18S rRNA [19]; in contrast, Chen et al. (2024) reported comparable limits of detection between LAMP and PCR for sheep *Sarcocystis* spp. when using the associated mitochondrial *cox*1 [20]. These discrepancies likely stem from the selected primers, as the efficiency of a LAMP assay depends critically on the specificity and binding efficiency of the utilized primers [12]. However, it is crucial to recognize the limitations of the LAMP assay. Problems like contamination, which may result in false-positive outcomes, difficulties in quantification leading to a decease in accuracy and reliability when compared with qPCR, its vulnerability to inhibitors in biological samples, and limited multiplexing ability (simultaneous detection of multiple target sequences in a single reaction is more challenging than in multiplex PCR) should not be ignored [11,18,21]. Therefore, additional optimization efforts are needed to enhance the consistency of LAMP assays and expand their application to different *Sarcocystis* species.

## 5. Conclusions

In this study, we developed the first rapid, visual, and field-deployable LAMP assay for the specific and sensitive detection of *S. miescheriana* and *S. suihominis* in domestic pigs, targeting the mitochondrial *cox*1 gene. The assay required only 40 min of incubation at 62 °C, demonstrated superior sensitivity to conventional PCR, and exhibited no cross-reactivity with related coccidia. Unlike PCR, the proposed LAMP method eliminates the need for costly thermocyclers or specialized personnel, making it ideal for primary laboratories, grassroots surveillance, and low-resource settings. This innovation offers significant potential for enhancing parasite control, thus preventing zoonotic transmission and protecting public health.

## Figures and Tables

**Table 1:** Species-discriminative LAMP primer sets targeting mitochondrial *cox*1 of *Sarcocystis* spp. in domestic pigs.

Target Species	Primer Name	Primer Length (nt)	Sequence (5′-3′)
*S. miescheriana*	F3	19	TGCTGCCACTTGGTTCAAC
	B3	18	AGTTGCCCGAACCCAGTA
	FIP	40	TCAGTGGTGCATACAGCGTCCCTCCACTCGTTCATTGCGG
	BIP	41	GGCGATGAATACGGAAGCCGTCGCTGCTAATACCGAGGAAC
*S. suihominis*	F3	20	CCATCGATGATCCTGGCAAT
	B3	18	CGGC AGCCATAACATCCA
	FIP	40	GTATCAACCTCGAGGCCGGCAGCCATACTCGGATCACTGG
	BIP	41	CCATTCCAACGGGCACGAAGAGGCGTATTTACTGCCCATGT

## Data Availability

All relevant data used in this study are contained within the manuscript, and the datasets used and/or analyzed during the current study are available from the corresponding author (J.H.) upon reasonable request. Nucleotide sequences of the mitochondrial *cox*1 (PX097380 and PX097381) of the samples have been deposited in GenBank.

## Abbreviations

The following abbreviations are used in this manuscript:

LAMP	Loop-mediated isothermal amplification
*cox*1	Cytochrome c oxidase subunit I gene
UV	Ultraviolet

## References

[1] Dubey J.P., Calero-Bernal R., Rosenthal B.M., Speer C.A., Fayer R. (2016). Sarcocystosis of Animals and Humans.

[2] Huang Z., Ye Y., Zhang H., Deng S., Tao J., Hu J., Yang Y. (2019). Morphological and molecular characterizations of *Sarcocystis miescheri* and *Sarcocystis suihominis* in domestic pigs (*Sus scrofa*) in China. Parasitol. Res..

[3] Helman E., Dellarupe A., Steffen K.D., Bernstein M., Moré G. (2024). Morphological and molecular characterization of *Sarcocystis* spp. in pigs (*Sus scrofa domestica*) from Argentina. Parasitol. Int..

[4] Golubkov V.I., Rybaltovskii D.V., Kislyakova Z.I. (1974). The source of infection for swine *Sarcocystis*. Veterinarya.

[5] Reiner G., Hepp S., Hertrampf B., Kliemt D., Mackenstedt U., Daugschies A., Zahner H. (2007). Genetic resistance to *Sarcocystis miescheriana* in pigs following experimental infection. Vet. Parasitol..

[6] Barrows P.L., Prestwood A.K., Green C.E. (1982). Experimental *Sarcocystis suicanis* infections: Disease in growing pigs. Am. J. Vet. Res..

[7] Piekarski G., Heydorn A.O., Aryeetey M.E., Hartlapp J.H., Kimmig P. (1978). Clinical, parasitological and serological investigations in sarcosporidiosis (*Sarcocystis suihominis*) of man (author’s transl). Immun. Infekt..

[8] Li Y., Lian Z. (1986). Study on man-pig cyclic infection of *Sarcocystis suihominis* found in Yunnan province, China. Acta Zool. Sin..

[9] Gjerde B. (2013). Phylogenetic relationships among *Sarcocystis* species in cervids, cattle and sheep inferred from the mitochondrial cytochrome c oxidase subunit I gene. Inter. J. Parasitol..

[10] Notomi T., Okayama H., Masubuchi H., Yonekawa T., Watanabe K., Amino N., Hase T. (2000). Loop-mediated isothermal amplification of DNA. Nucleic Acids Res..

[11] Nzelu C.O., Kato H., Peters N.C. (2019). Loop-mediated isothermal amplification (LAMP): An advanced molecular point-of-care technique for the detection of *Leishmania* infection. PLoS Negl. Trop. Dis..

[12] Obande G.A., Banga Singh K.K. (2020). Current and future perspectives on isothermal nucleic acid amplification technologies for diagnosing infections. Infect. Drug Resist..

[13] Gjerde B. (2014). *Sarcocystis* species in red deer revisited: With a re-description of two known species as *Sarcocystis elongata* n. sp. and *Sarcocystis truncata* n. Sp. based on mitochondrial cox1 sequences. Parasitology.

[14] da Rosa G., Roman I.J., Gressler L.T., Cargnelutti J.F., Vogel F.S.F. (2024). Molecular identification of *Sarcocystis* species in wild boar (*Sus scrofa*) and pigs (*Sus scrofa domesticus*) in Brazil. Vet. Parasitol. Reg. Stud. Rep..

[15] Imre K., Sala C., Morar A., Imre M., Ciontu C., Chisăliță I., Dudu A., Matei M., Dărăbuș G. (2017). Occurrence and first molecular characterization of *Sarcocystis* spp. in wild boars (*Sus scrofa*) and domestic pigs (*Sus scrofa domesticus*) in Romania: Public health significance of the isolates. Acta. Trop..

[16] Zainalabidin F.A., Noorazmi M.S., Bakri W.N., Sathaya G., Ismail M.I. (2017). Prevalence of muscular sarcosporidiosis in slaughtered domestic Pigs in Perak, Peninsular Malaysia. Trop. Life. Sci. Res..

[17] Pacifico L., Rubiola S., Buono F., Sgadari M., D’Alessio N., Scarcelli S., Sgroi G., Buglione M., Chiesa F., Restucci B. (2023). Molecular differentiation of *Sarcocystis miescheriana* and *Sarcocystis suihominis* using a new multiplex PCR targeting the mtDNA cox1 gene in wild boars in southern Italy. Res. Vet. Sci..

[18] Li Y., Fan P., Zhou S., Zhang L. (2017). Loop-mediated isothermal amplification (LAMP): A novel rapid detection platform for pathogens. Microb. Pathog..

[19] Liu D., Zhuo Z., Hou Z., Tao J., Xu J. (2022). Establishment and application of a loop-mediated isothermal amplification assay for detection of *Sarcocystis suihominis* in pigs. Chin. Vet. Sci..

[20] Chen Y., Peng J., Zhu Z., Zhang W., Wang L., Xu J., Liu Q., Liu J. (2024). Development of a highly specific LAMP assay for detection of *Sarcocystis tenella* and *Sarcocystis gigantea* in sheep. Parasitol. Res..

[21] Wong Y.P., Othman S., Lau Y.L., Radu S., Chee H.Y. (2018). Loop-mediated isothermal amplification (LAMP): A versatile technique for detection of micro-organisms. J. Appl. Microbiol..

